# BCMA/CS1双靶点CAR-T细胞治疗多发性骨髓瘤并发血小板减少1例报告并文献复习

**DOI:** 10.3760/cma.j.cn121090-20241202-00524

**Published:** 2024-12

**Authors:** 沛儒 李, 莹莹 李, 孟仪 都, 慧雯 江, 恒 梅

**Affiliations:** 华中科技大学同济医学院附属协和医院血液科，武汉 430022 Institute of Hematology, Union Hospital, Tongji Medical College, Huazhong University of Science and Technology, Wuhan 430022, China

**Keywords:** 多发性骨髓瘤, 血小板减少, 嵌合抗原受体T细胞治疗, B细胞成熟抗原, Multiple myeloma, Thrombocytopenia, Chimeric antigen receptor T cell therapy, B-cell maturation antigen

## Abstract

**目的:**

探讨BCMA/CS1双靶点嵌合抗原受体T细胞（CAR-T细胞）治疗多发性骨髓瘤后并发血小板减少的临床表现和诊疗策略。

**方法:**

回顾性收集2021年6月于华中科技大学同济医学院附属协和医院血液科接受BCMA/CS1双靶点CAR-T细胞治疗的1例多发性骨髓瘤患者的临床资料并进行分析。以“嵌合抗原受体T细胞治疗”、“血小板减少”、“chimeric antigen receptor-T cell therapy”、“CAR-T”、“thrombocytopenia”、“BCMA”、“CS1”为检索词，检索自建库至2024年11月中文数据库（中国知网数据库、万方数据库）及Pubmed数据库中的文献并进行文献复习。

**结果:**

患者，男，72岁，于当地医院诊断为轻链κ型多发性骨髓瘤（R-ISS Ⅱ期）。患者治疗周期较长，多次调整化疗方案后疾病控制不佳。经筛选患者符合BCMA/CS1双靶点CAR-T细胞临床试验（NCT04662099）纳入标准，回输低剂量CAR-T细胞2个月后患者达到完全缓解。CAR-T细胞回输后第8天患者发生3级细胞因子释放综合征，随后出现严重消化道出血症状及持续≥3级血小板减少，伴促血小板生成素水平显著升高，骨髓细胞学未见巨核细胞。患者接受利妥昔单抗治疗后血小板逐渐回升，疗效显著。后因患者持续营养不良伴消瘦，转入消化科治疗后失访。文献检索共检索到相关英文文献24篇，中文文献8篇。

**结论:**

CAR-T细胞治疗相关血小板减少表现为血小板减少和巨核细胞生成障碍，持续性血小板减少增加出血风险，应用CD20单抗及支持治疗可有效改善血小板减少。

## Abstract (en)

**Objective:**

To investigate the clinical manifestations, diagnostic and therapeutic strategies, for thrombocytopenia after BCMA/CS1 bispecific chimeric antigen receptor T-cell（CAR-T cell）therapy in patients with multiple myeloma.

**Methods:**

Retrospective collection and analysis were conducted on the clinical data of a patient with multiple myeloma who received BCMA/CS1 bispecific CAR-T cell therapy in the Hematology Department of Union Hospital, Tongji Medical College, Huazhong University of Science and Technology, in June 2021. To review the literature, Chinese databases（CNKI, Wanfang Database）and PubMed were searched from their inception up to November 2024 using the keywords “chimeric antigen receptor T-cell therapy”, “thrombocytopenia”, “CAR-T”, “BCMA”, and “CS1”.

**Results:**

A 72-year-old male patient was diagnosed with light chain κ-type multiple myeloma（R-ISS stage Ⅱ）in a local hospital and had a prolonged treatment course with poor disease control despite multiple adjustments of chemotherapy regimens. The patient met the inclusion criteria for the BCMA/CS1 dual-target CAR-T cell clinical trial（NCT04662099）and achieved complete remission two months after receiving a low dose of CAR-T cells. On the 8th day after CAR-T cell infusion, the patient developed grade 3 cytokine release syndrome, followed by severe gastrointestinal bleeding and persistent grade ≥3 thrombocytopenia, accompanied by a significant increase in thrombopoietin levels, with no megakaryocytes observed in bone marrow cytology. After treatment with rituximab, significant improvement was observed with platelet count gradually increased. However, the patient experienced persistent malnutrition and cachexia, and transferred to the gastroenterology department, after which follow-up was lost. A total of 24 relevant English articles and 8 Chinese articles were retrieved from the literature search.

**Conclusion:**

CAR-T cell therapy associated thrombocytopenia is characterized by thrombocytopenia and megakaryocyte production disorders. Persistent thrombocytopenia increases the risk of bleeding, and the use of CD20 monoclonal antibodies and supportive therapy can effectively improve thrombocytopenia.

多发性骨髓瘤（multiple myeloma，MM）是由克隆性浆细胞异常增殖引起的一种恶性疾病，至今仍不可治愈[1]–[3]。嵌合抗原受体T细胞（chimeric antigen receptor-T cell，CAR-T细胞）疗法在MM等复发/难治性血液肿瘤治疗领域取得了突破性进展[4]–[6]，国家药品监督管理局已批准3款靶向B细胞成熟抗原（B cell maturation antigen，BCMA）的CAR-T细胞产品（伊基奥仑赛注射液、泽沃基奥仑赛注射液、西达基奥仑赛注射液）用于治疗复发/难治性MM。近年来，以BCMA和CS1为靶点的CAR-T细胞疗法因其靶向性更广和克服单靶点耐药作用引起广泛关注，有效率高达81％[7]–[12]。然而，CAR-T细胞治疗相关毒性不可忽视，除细胞因子释放综合征（cytokine release syndrome，CRS）和免疫效应细胞相关神经毒性综合征外，免疫效应细胞相关血液毒性也是影响其安全性的重要挑战[7],[13]。80％以上接受CAR-T细胞治疗的患者出现3级及以上血液学毒性[7],[14]，其中血小板减少发生率为30％~63％[15]。目前尚无统一的CAR-T细胞相关血小板减少临床管理指南，潜在机制有待进一步探索。本文报道一例MM患者在接受BCMA/CS1双靶点CAR-T细胞治疗后出现血小板减少的病例，并进行文献复习。

## 病例资料

患者，男，72岁，2016年6月无明显诱因出现右侧髋关节疼痛、腰痛及双下肢乏力。腰椎增强计算机断层扫描（CT）：L3椎体及附件骨质破坏；骨髓细胞学：原始幼稚浆细胞34％，荧光原位杂交示IGH易位t（11;14）（q13;q32）；血清游离轻链和24 h尿蛋白电泳结果不详，当地医院诊断为κ轻链型多发性骨髓瘤（R-ISS Ⅱ期），行腰椎肿瘤切除+椎弓根内固定术，应用TD方案（沙利度胺+地塞米松）1个周期、BD方案（硼替佐米+地塞米松）19个周期、RD方案（来那度胺+地塞米松）维持治疗1年余。2020年9月因疾病复发于外院换用伊沙佐米治疗，后因不良反应较重停用骨髓瘤治疗药物。2021年5月23日就诊于我科，查体：左侧颈部可见3 cm×1 cm长方形包块，色红，右肺可闻及细湿啰音。实验室检查：WBC 6.53×10^9^/L，HGB 92 g/L，PLT 198×10^9^/L，中性粒细胞绝对值4.03×10^9^/L；血清游离轻链：κ 822.5 mg/L，κ/λ 47.27，血清免疫固定电泳和尿本周蛋白电泳提示κ型M蛋白血症；骨髓流式细胞术微小残留病：原始-前体区域细胞约占有核细胞的1％，CD34^+^细胞约占0.06％，髓系区域可见约8.7％的CD38^bri^CD138^+^CD19^-^cKappa^+^细胞，部分表达CD56，考虑为免疫表型异常的残留细胞；正电子发射断层显像/X线计算机体层成像（PET/CT）：颅骨、双侧锁骨、右侧肱骨、双侧肩胛骨，双侧多发肋骨、胸骨、多发椎体、双侧股骨头、骨盆及探测范围内双侧股骨多发稍低密度影，代谢不高，部分较前为新发；右颈部1B区团块，代谢异常增高，较前为新发，考虑为MM浸润，总体较前进展。提示患者为复发/难治性MM。经筛选，患者符合BCMA/CS1双靶点CAR-T细胞临床试验（NCT04662099）纳入标准，签署知情同意书后，采集自体单个核细胞用于制备CAR-T细胞。2021年6月行预处理化疗（氟达拉滨30 mg/m^2^第1～3天，环磷酰胺250 mg/m^2^第1～3天）。回输前PLT 71×10^9^/L，余实验室检查结果见表1。荧光原位杂交示IGH易位t（11;14）（q13;q32），融合信号细胞占19％；血清游离轻链：κ 785 mg/L、κ/λ 65.42，血清免疫固定电泳和尿本周蛋白电泳示κ型M蛋白血症。2021年6月回输低剂量CAR-T细胞0.75×10^6^个/kg。回输后第2天出现粒细胞缺乏，予美罗培南联合卡泊芬净抗感染治疗。第8～10天患者持续发热38 °C左右，血压下降，血氧饱和度不能维持；WBC 0.84×10^9^/L，HGB 54 g/L，PLT 44×10^9^/L，中性粒细胞绝对值0.15×10^9^/L；铁蛋白1 034.2 µg/ml，C反应蛋白101.75 mg/L，IL-6 61.46 pg/ml，IL-10 29.88 pg/ml。根据美国移植与细胞治疗学会分级标准诊断为3级CRS，予面罩吸氧，去甲肾上腺素升压，甲泼尼龙拮抗CRS，加用替考拉宁抗感染，患者体温恢复正常。第14天PLT 24×10^9^/L；骨髓细胞学示幼稚浆细胞占6％，未见巨核细胞；荧光原位杂交未见IGH易位t（11;14）（q13;q32）异常信号细胞；骨髓流式细胞术CAR-T细胞比例约88.4％；外周血流式细胞术CAR-T细胞比例约84.3％；血清游离轻链：κ 126 mg/L，λ 5.2 mg/L，κ/λ 24.231；尿游离轻链：κ 2 150 mg/L，λ 18.7 mg/L；血清免疫固定电泳和尿本周蛋白电泳结果仍示κ型M蛋白血症。第15天患者再次出现发热，体温37.8 °C，铁蛋白6 111 µg/ml，IL-6 193.24 pg/ml，IL-10 229.82 pg/ml，PLT 44×10^9^/L，纤维蛋白原1.07 g/L。予托珠单抗及甲泼尼龙拮抗CRS，予人重组血小板生成素、补充纤维蛋白原，患者体温恢复正常，血小板和纤维蛋白原水平无明显好转。第21天骨髓细胞形态学未见幼稚浆细胞。第22～24天PLT降至19×10^9^/L，纤维蛋白原持续低于1.5 g/L，其余凝血指标无明显异常；患者出现消化道出血，间断呕血、便血，总出血量约1 200 ml，予输注红细胞、血小板、纤维蛋白原，酚磺乙胺、醋酸奥曲肽、氨基己酸、垂体后叶素止血治疗后消化道出血症状好转。CAR-T细胞回输后1个月骨髓细胞学未见幼稚浆细胞，未见巨核细胞，血清免疫固定电泳阴性，尿本周蛋白电泳结果仍示κ型M蛋白血症；血清游离轻链恢复至正常水平，尿游离轻链：κ 17.8 mg/L，λ 7.57 mg/L，提示患者达到非常好的部分缓解。CAR-T细胞回输后1个月内间断补充纤维蛋白原8 g，可恢复至1.5 g/L及以上。共输注血小板12人份并持续予人重组血小板生成素，PLT仍低于50×10^9^/L，后改用阿伐曲泊帕，治疗仍无效。查血浆促血小板生成素1 280 pg/ml，血小板特异性抗体抗GPⅢa阳性，自身免疫性疾病全套阴性，骨髓未见噬血现象。予利妥昔单抗治疗后，PLT恢复至50×10^9^/L以上，血小板特异性抗体检测转为阴性。回输后2个月外周血和骨髓CAR-T细胞持续存在，骨髓细胞学未见幼稚浆细胞，巨核细胞恢复，血清和尿免疫固定电泳、游离轻链均恢复正常，PLT维持在70×10^9^/L左右，纤维蛋白原维持在1.5 g/L及以上，其余凝血指标正常，患者完全缓解。后因患者持续营养不良伴消瘦，转入消化科治疗后失访。

**表1:** 本例患者CAR-T细胞回输后相关实验室指标变化

实验室指标	嵌合抗原受体T细胞回输后时间							
	0 d	8 d	14 d	15 d	21 d	23 d	1个月	2个月
WBC（×10^9^/L）	0.58	0.84	6.01	9.55	2.16	1.75	3.56	3.92
HGB（g/L）	70	54	69	55	59	63	109	76
PLT（×10^9^/L）	71	44	24	44	31	19	27	64
C-反应蛋白（mg/L）	15.20	101.75	34.06	33.34	12.59	21.48	6.66	–
铁蛋白（µg/ml）	714.0	1034.2	4369.0	6111.0	2766.5	2120.1	117.4	–
IL-6（pg/ml）	18.19	61.46	172.07	193.24	334.14	599.72	526.32	–
IL-10（pg/ml）	4.50	29.88	117.00	229.82	3.96	3.96	9.27	–
血清游离κ轻链（mg/L）	785.0	–	126.0	–	–	–	6.2	6.2
血清游离λ轻链（mg/L）	12.00	–	5.20	–	–	–	5.20	7.31
尿游离κ轻链g/L）	–	–	2150.0	–	–	–	17.8	0
尿游离λ轻链（g/L）	–	–	18.70	–	–	–	7.57	0
骨髓幼稚浆细胞（％）	–	18	6	–	0	–	0	0
骨髓巨核细胞（个）	–	3	0	–	0	–	0	6
外周血CAR-T细胞（％）	–	2.4	84.3	85.9	50.4	–	12.0	4.8
骨髓CAR-T细胞（％）	–	3.0	88.4	–	63.4	–	16.8	6.0

**注** –：无数据

## 讨论及文献复习

本病例为复发/难治性MM老年男性患者，因合并心血管疾病，予低剂量CAR-T细胞回输，治疗2个月后达到完全缓解。根据不良事件常用术语标准，该患者在CAR-T细胞回输后出现4级全血细胞减少，WBC和HGB在1个月内恢复正常，但≥3级血小板减少仍持续存在，伴促血小板生成素水平显著升高，骨髓细胞学未见巨核细胞，血小板特异性抗体抗GPⅢa阳性，自身免疫性疾病全套阴性，提示血小板减少可能由CAR-T细胞治疗后的免疫效应介导。

根据既往研究，CAR-T细胞相关血小板减少呈双相变化，早期（CAR-T细胞回输后28 d内）发生率为31％～79％，其中24％～60％发生≥3级血小板减少[16]。此外，21％～29％发生≥3级的晚期血小板减少（28 d后）[17]–[22]。高龄、既往多线治疗（≥3线）、骨髓浸润程度、基线细胞减少、基线炎症状态、异基因造血干细胞移植、重度CRS和神经系统不良反应是血小板减少的危险因素[15]–[16],[20],[23]–[25]。目前针对CAR-T细胞治疗后血小板减少的发病机制尚缺乏深入研究，可能涉及炎症因子、骨髓微环境及自身免疫反应等。CRS发生时，大量IL-6、TNF-α及干扰素-γ等可破坏骨髓微环境，引起骨髓抑制，进而影响巨核细胞生成和血小板释放；大量细胞因子损伤内皮屏障，引发凝血级联反应，增加血小板的消耗和破坏；同时，活化的CAR-T细胞可能激活B细胞，产生过多的血小板特异性抗体，导致巨核细胞和血小板破坏增多[26]–[29]。此外，既往多线治疗、回输前的预处理化疗及CAR-T细胞的脱靶效应都可能加重血小板减少的程度[30]。

本病例中，由于重度CRS的发生，骨髓中大量细胞因子浸润，影响骨髓造血能力。同时，过多的血小板特异性抗体对血小板及巨核细胞的破坏导致重度血小板减少并恢复延迟，促血小板生成素治疗无效，CD20单抗应用后血小板恢复，疗效显著。
